# Neuroimmune Activation Drives Multiple Brain States

**DOI:** 10.3389/fnsys.2018.00039

**Published:** 2018-08-29

**Authors:** Daria Tchessalova, Caitlin Kelly Posillico, Natalie Celia Tronson

**Affiliations:** ^1^Neuroscience Graduate Program, School of Medicine, University of Michigan, Ann Arbor, MI, United States; ^2^Department of Psychology, University of Michigan, Ann Arbor, MI, United States

**Keywords:** neuroimmune, learning and memory, vulnerability, persistent changes, cognition, brain states

## Abstract

Neuroimmune signaling is increasingly identified as a critical component of neuronal processes underlying memory, emotion and cognition. The interactions of microglia and astrocytes with neurons and synapses, and the individual cytokines and immune signaling molecules that mediate these interactions are a current focus of much research. Here, we discuss neuroimmune activation as a mechanism triggering different states that modulate cognitive and affective processes to allow for appropriate behavior during and after illness or injury. We propose that these states lie on a continuum from a naïve homeostatic baseline state in the absence of stimulation, to acute neuroimmune activity and chronic activation. Importantly, consequences of illness or injury including cognitive deficits and mood impairments can persist long after resolution of immune signaling. This suggests that neuroimmune activation also results in an enduring shift in the homeostatic baseline state with long lasting consequences for neural function and behavior. Such different states can be identified in a multidimensional way, using patterns of cytokine and glial activation, behavioral and cognitive changes, and epigenetic signatures. Identifying distinct neuroimmune states and their consequences for neural function will provide a framework for predicting vulnerability to disorders of memory, cognition and emotion both during and long after recovery from illness.

## Introduction

Behavioral states or brain states are defined as co-ordinated patterns of activity in the brain. Whether that state is a “feeling” (philosophical, Oosterwijk et al., 2012), a systems/circuit activation (Quilichini and Bernard, 2012), or a pattern of kinase activity (Tronson et al., 2012; Mucic et al., 2015), brain states can be thought of as a snapshot of what is happening in the brain in that moment. By adjusting to the precise current conditions of the animal’s environment, changes in state are essential for efficient processing of information relevant to that place and time, and as such, are accompanied by changes in information processing in the hippocampus (Anderson et al., 2014) and cortex (Tsuno and Mori, 2009). In this review, we discuss neuroimmune activity as a continuum of states—including a baseline homeostatic state, acute activation, chronic low-grade activation and a “vulnerable” state that persists long after a major illness or injury—that interact with neural function and cognition.

## The Immune System: A Primer

In the periphery, the immune system is divided into two interrelated but separable systems—the innate immune system and the adaptive immune system. The innate immune system enacts the rapid response to infectious agents and injury via specialized receptors that recognize viruses (double stranded RNA), bacteria (e.g., lipopolysaccharides, LPS), or nuclear and cytosolic proteins that are released during cellular damage. The innate immune response is characterized by an expansion and subsequent resolution of cytokine production. The initial response includes cytokines (e.g., interleukins IL-1β, IL-6; tumor necrosis factor α (TNFα), interferon γ (IFN γ)) and chemokines (e.g., CX3CL1, CSF1-3) that recruit additional immune cells, and increase transcription and production of cytokines. This also increases production of regulatory cytokines (e.g., IL-4, IL-10) that, together with intracellular signaling proteins (e.g., SOCS, suppressor of cytokine signaling; PIAS1) suppress cytokine production and immune cell recruitment, thereby resolving the immune response (Wang and Campbell, 2002; Colton, 2009; Schmitz et al., 2011; Becher et al., 2017; Prieto and Cotman, 2017). The adaptive immune system is, in turn, activated by the innate immune response, with a slower response and resulting in the generation of antibodies to the infectious pathogen (Janeway, 2001).

Neuroimmune processes are activated during peripheral illness due to vagal nerve activity and immune signals that cross the blood brain barrier (McCusker and Kelley, 2013), and as a consequence of neural injury or infection. The neuroimmune response in the brain is predominantly considered an innate immune response, however components of the adaptive immune system in the meningeal compartment, including T-cells, are critical for normal neural function (Kipnis et al., 2012), cognitive function, and production of cytokines in the brain during illness or injury (Marin and Kipnis, 2017). Acute activation of neuroimmune processes function to both heal neural injury and attack infection in the brain (Kreutzberg, 1996; Berczi et al., 2009; Kawabori and Yenari, 2015; DiSabato et al., 2016; Sochocka et al., 2017), and as an important adaptive process, triggering sickness behaviors and physiological responses that allow the peripheral immune system to perform at optimal levels (McCusker and Kelley, 2013).

## Naïve Homeostatic Baseline State

Although we commonly refer to illness or injury as triggering “activation” of the immune and neuroimmune systems, the naïve homeostatic baseline is not the absence of neuroimmune signaling or activity. Instead, astrocytes and microglia constantly interact with neurons and play active roles in regulation of neural function and synaptic plasticity. Astrocytes provide energy and regulate glutamate during synaptic transmission and plasticity (Suzuki et al., 2011; Gold, 2014; Nortley and Attwell, 2017; Alberini et al., 2018). Microglia provide continuous surveillance, synaptic pruning, and regulation of synaptic plasticity via complement and cytokine interactions (Nimmerjahn et al., 2005; Wu et al., 2015; Lenz and Nelson, 2018; Pósfai et al., 2018). Importantly, neurons produce and have receptors for “immune” proteins including cytokines, chemokines and complement, thereby providing a basis for ongoing communication with glial cells (Freidin et al., 1992; Veerhuis et al., 2011; McCusker and Kelley, 2013; Paolicelli et al., 2014).

Cytokines and chemokines are also produced during this baseline state and during non-inflammatory stimulation such as learning and induction of long-term plasticity (Jankowsky et al., 2000; del Rey et al., 2013). These cytokines play both permissive (e.g, IL-1β; (Goshen et al., 2007); IL-4 (Gadani et al., 2012)) and suppressive (e.g., IL-1β (Avital et al., 2003; Goshen et al., 2007); IL-6 (Balschun et al., 2004)) roles in synaptic plasticity and memory formation during adulthood. Rather than the (neuro)immune system as existing as either “off” or “on,” it is more accurately conceptualized as a continuum of activity from a homeostatic baseline state, through undetectable response to cellular stress, up through low-grade activity, and occasionally a fully active disease or acute inflammatory state (e.g., Chovatiya and Medzhitov, 2014; Marques et al., 2016). In accordance with this view and consistent with the growing evidence for a critical role of immune cells and signaling in normal memory, affective and cognitive processes, the neuroimmune system is not inactive in the absence of inflammatory stimulation, but instead engages in ongoing interactions with synapses, neurons and circuits in the brain (Capuron and Miller, 2011; McCusker and Kelley, 2013; Donzis and Tronson, 2014; Wu et al., 2015; Tronson and Collette, 2017; Dantzer, 2018).

Nevertheless, the levels of cytokines (Erickson and Banks, 2011; Biesmans et al., 2013; Speirs and Tronson, 2018) and cytokine expression (Skelly et al., 2013) in the brain during non-stimulated conditions are very low, often at concentrations that are barely detectable (Erickson and Banks, 2011; Biesmans et al., 2013). During activation by LPS, for example, whereas cytokines in serum can be hundreds or thousands fold higher than baseline, in the brain, these changes are limited to 1.5–10 fold range (Erickson and Banks, 2011; Biesmans et al., 2013; Speirs and Tronson, 2018) with occasional chemokines showing hundreds of fold increases (e.g., CXCL10, CSF3; Speirs and Tronson, 2018). The difficulty in measuring immune signaling in the brain (and in the periphery, Chovatiya and Medzhitov, 2014) in the baseline state therefore makes it difficult to assess the precise roles in these baseline processes.

## Acute Neuroimmune Activation State

Acute immune activation, whether by illness, injury, or experimental administration of LPS or other immune trigger, results in the induction of broad networks of cytokines and immune molecules, including complement, IL-1β, IL-6 and TNFα, followed by the regulatory cytokines (IL-10, IL-4) and downstream signaling pathways (Donzis and Tronson, 2014).

That the activation of the neuroimmune system drives a unique brain and behavioral state is clear from the distinct consequences of illness on behavior, cognition, emotion and learning and memory (Dantzer et al., 1998; Yirmiya and Goshen, 2011; Donzis and Tronson, 2014). The roles of cytokines and immune molecules in the modulation of memory has been a particular point of recent interest (Pugh et al., 1998; Marin and Kipnis, 2013; del Rey et al., 2013). Elevations of IL-1β results in disruption of memory and synaptic plasticity (Cunningham et al., 1996; Ross et al., 2003; Goshen et al., 2007; Gonzalez et al., 2013); and IL-6 activation acts as a brake on plasticity (Balschun et al., 2004; Sparkman et al., 2006). In addition to cytokines, other immune signaling pathways including complement signaling are critical for neuroimmune activation (Jacob et al., 2007), physiological effects including fever (Boos et al., 2005) and synaptic plasticity (Boulanger, 2009; Bitzer-Quintero and González-Burgos, 2012; Zhang et al., 2014). For example, the complement proteins are produced by neurons and microglia during infection, triggering microglial infiltration and activation, loss of hippocampal synapses and spatial memory impairments during West Nile Virus infection, effects prevented by knockout of the C3 or blockade of C1qa complement production (Vasek et al., 2016). Immune signaling molecules are therefore a defining feature, and drive outcomes of, the acute neuroimmune activation state.

Morphological and transcriptional changes of microglia and astrocytes are also indicative of neuroimmune activation. Activated microglia show a shift in morphology from a ramified, surveying state to an ameboid state by withdrawing long, thin processes and extending short, thick, phagocytic processes towards the source (Stence et al., 2001). Similarly, astrocytes have several distinct “reactive” transcriptional profiles in response to immune stimulation (Zamanian et al., 2012; Liddelow and Barres, 2017). Activated microglia are a primary source of cytokine and chemokine production during immune events (Colonna and Butovsky, 2017), and thereby alter neuronal-glia communication (Tremblay et al., 2011; Paolicelli et al., 2014). Indeed, inhibition of microglial activity prevents immune challenge-induced impairments in memory, suggesting a requirement for these neuroimmune cells in cognitive deficits during acute neuroimmune activity (Yang et al., 2015; Vasek et al., 2016; Wang et al., 2016; Wadhwa et al., 2017).

The role of a small number of cytokines, notably IL-1β, IL-6 and IL-4, in modulating behavior, cognition and memory processes is well established. Nevertheless, the functional role of specific immune signaling molecules depends on the current immune milieu and the specific immune challenge. For example, IFNγ can enhance immune signaling as observed in models of cerebral malaria and after LPS, or downregulate inflammatory responses in the brain, as in experimental allergic encephalomyelitis (Heremans et al., 1989). Similarly, the role of IL-1 in the brain differs between neurodegenerative and neuroinflammatory states, and depends on the subsets of immune cells and cytokines present (Becher et al., 2017). The precise activation state and transcriptional profile of immune cells also differs depending on the type of immune stimulation, with different immune triggers resulting in different patterns of gene expression and cytokine signaling (Chovatiya and Medzhitov, 2014; Becher et al., 2017; Prieto and Cotman, 2017). This means that in considering the individual roles of cytokines in modulation of neural processes, we also need to bear in mind the broader context of what other cytokines and immune cells are concurrently present.

A special subset of neuroimmune activation is chronic inflammation. As with acute immune activation, chronic inflammation is defined by persistent activation of cytokines and glial cells, and results in changes in behavior and neural function. In models of sepsis, for example, microglial activation persists for weeks or months after surgery, presumably contributing to persistent cognitive deficits (Weberpals et al., 2009; Singer et al., 2016; Olivieri et al., 2018). Similarly, after traumatic brain injury, there is long-term inflammatory activity (Gentleman et al., 2004). In patients of arthritis and periodontal disease, chronic low-grade inflammation is associated with cognitive deficits, depression and increased risk for Alzheimer’s disease (Kamer et al., 2008; Chou et al., 2016; Simos et al., 2016), and animal models suggest a causal link between chronic inflammatory conditions and altered cognitive function and increased depression-like behaviors (Brown et al., 2018; Ding et al., 2018). Long-term increases in cytokine levels in the brain likely result in a notably different cytokine network and therefore a brain states that is distinct from acute neuroimmune activation states.

Collectively, these studies point to neuroimmune activation as being crucial for determining the profile and function of neural activity that mediates changes in behavior, cognition, affect and memory. Furthermore, the importance of specific patterns of immune signaling on cognition and behavior suggests that acute neuroimmune activation is not one, but multiple distinct brain state, depending on the specific immune trigger and, as described below, previous immune experience.

## Persistent Changes in Brain State After Immune Challenge

A defining feature of the peripheral immune system is that acute activation results in permanent changes to immune function that persist after resolution of inflammatory signaling. The best-known example of this is the adaptive immune system, which becomes able to produce new antibodies after exposure to a pathogen (Janeway, 2001). Independently, the innate immune system also shows “trained” immunity, in which a transient immune challenge results in increased responsiveness to subsequent immune stimuli (Netea and van der Meer, 2017). Such long-lasting changes may be thought of as a distinct, shifted baseline state that results in functional adaptation for subsequent illnesses. If the innate immune system in the periphery encodes experiences as long-lasting changes in function, it is likely that persistent changes are also encoded in the neuroimmune system.

Permanent changes in neuroimmune function have been observed after prenatal or early life immune challenge in and effect termed “microglia priming.” Here maternal immune challenge during pregnancy or early life immune challenge result in increased responsiveness of microglia to immune challenge during adulthood (Schwarz and Bilbo, 2012; Bilbo, 2013; Haley et al., 2017). More recently, several studies demonstrate similar priming effects after immune challenge in adults, with changes in microglial and astrocytic function and behavior (Fenn et al., 2014; Norden et al., 2015; Muccigrosso et al., 2016; Liddelow and Barres, 2017; Olivieri et al., 2018; Wendeln et al., 2018). Importantly, these effects persist even when the pathogenic process is eliminated long before. By altering interactions between neurons and glial cells and the quantity and specific patterns of cytokines produced (ŠiŠková and Tremblay, 2013; Wendeln et al., 2018), prior illness has a lasting impact on the effect of subsequent acute immune challenge. For example, an LPS challenge results in altered gene expression and increased cytokines in response to stroke at least 6 months later, suggesting persistent changes in neuroimmune function even when animals have shown a full recovery (Wendeln et al., 2018).

Long-lasting functional changes are mediated by epigenetic modifications triggered by immune activation. In the periphery, methylation of histone H3 mediates increased innate immune responses (Kleinnijenhuis et al., 2012). Similarly, in the brain, acetylation and methylation of H3 are associated with altered astrocyte and microglial function after immune challenge (Schaafsma et al., 2015; Haley et al., 2017; Wendeln et al., 2018). Together, these findings demonstrate that a prior immune challenge causes persistent changes in neuroimmune function, or neuroimmune “training,” and results in vulnerability to later immune events.

One question arising from these lasting changes in neuroimmune function is whether there are also changes in the homeostatic baseline state of the brain. If so, transient illness may cause not only the initial acute immune state, but also a new permanently altered brain state. There are several pieces of evidence that support changes in brain function that persist long after the resolution of immune challenge. In addition to altered neuroimmune reactivity, peripheral illness can result in decreased neuronal connectivity, synaptic spines and plasticity (Kondo et al., 2011; Maggio et al., 2013; Huerta et al., 2016). Alternatively, long-lasting increases in permeability of blood-brain barrier may also contribute to persistent changes in baseline homeostatic state of the brain (Saunders et al., 2008; Strbian et al., 2008). Consistent with these data, we have recently observed memory impairments that emerge and persist months after an immune challenge (Tchessalova and Tronson, 2018). Changes in neuronal connectivity, neuroimmune function, and their interactions mediate what and how information is processed, and contribute to persistent changes in cognitive function, mood and memory observed after illness or injury.

**Figure 1:**
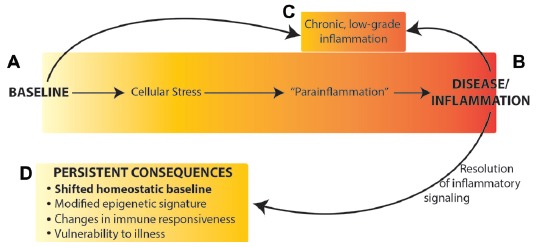
Neuroimmune activation occurs along a continuum from the naïve (homeostatic) baseline **(A)**, to an active inflammatory state **(B)** or chronic inflammation **(C)**. We propose that resolution of inflammatory signaling does not result in return to the original baseline, but rather results in persistently altered homeostatic baseline **(D)** mediated by epigenetic changes in the brain. Figure adapted from Chovatiya and Medzhitov (2014).

Collectively, these data suggest that rather than returning to the original homeostatic baseline state after resolution of an immune challenge, the brain reaches a new homeostatic state with different neuronal connections, a “trained” neuroimmune system, and epigenetic modifications that lead to dysregulated gene expression (Figure 1). A shift in baseline state and neuronal connectivity could result in changes to neural and neuroimmune responses to environmental stimuli, including immune and other sensory information, and thus altered cognitive processes and vulnerability to memory, affective and neurodegenerative disorders.

## Discussion and Conclusions

The work reviewed here clearly demonstrates that neuroimmune activation modulates neural and cognitive function both acutely and with effects that persist long after recovery from illness and the resolution of a transient immune response. There is growing evidence for the role of neuroimmune signaling in both normal and pathological memory, cognitive and affective processes. In particular, the role of individual cytokines in the regulation of synaptic plasticity and memory, and the dysregulation during an immune response, has been highlighted by recent research. Importantly, activation of neuroimmune signaling is more complex than single effectors. Instead, the neuroimmune response includes activation of glial cells, cytokines and many other immune signaling molecules in a co-ordinated manner (Schmitz et al., 2011; Becher et al., 2017; Prieto and Cotman, 2017), which in turn causes a distinct set of regulatory cytokines and signaling molecules that mediate resolution of the response (McCusker and Kelley, 2013). This activation and resolution cycle further results in long lasting epigenetic modifications and changes in neuronal connectivity that persist and modulate cognitive function and behavior long after the end of the immune challenge. We therefore propose that neuroimmune activation can be viewed as a continuum of states in the brain: from acute or chronic activation, to resolution, to persistent changes in baseline homeostatic state(s), each with distinctive patterns of glial cell activation, cytokine and immune signaling, epigenetic modifications, and behavioral change (Figure 1).

### How to Define Neuroimmune “States”?

An important unresolved question is how to define neuroimmune states? In general, neuroimmune activation is commonly defined by behavioral changes, by the activation of microglia, or by increased level or expression of immune molecules, in particular cytokines and chemokines (Dantzer et al., 1998; McCusker and Kelley, 2013; del Rey et al., 2013; Becher et al., 2017). Behavioral changes, notably sickness behaviors and associated febrile response and weight loss, are commonly used to assess immune (and neuroimmune) activation. Yet sickness responses are not specific to immune trigger—as noted by McCusker and Kelley (2013), “symptoms are commonly expressed by sick animals despite the broad spectrum of possible pathogens.” Therefore, sickness behaviors are not sensitive enough to the precise neuroimmune state of the brain beyond “on” and “off”. Morphological changes of microglia are useful for defining when the neuroimmune system is active but is likely not sufficient for identifying changes in homeostatic baseline or other long lasting, low-grade changes. Alternatively, identifying the precise patterns of cytokines expressed and neuroimmune cells recruited after an immune challenge allows for a detailed definition of different active states. The two disadvantages of this being the sole approach to identifying state, however, are: (1) that levels of cytokines at baseline are very low, thus it is difficult to clearly identify what is happening in the absence of acute neuroimmune activation; and (2) if broad patterns of cytokines define a state it is not sufficient to simply measure a small number of cytokines, and this approach rapidly becomes cumbersome and expensive. Thus, in order to use cytokines and immune signaling to define states, it will be critical to identify a smaller cluster of key proteins distinct for each state. Importantly, behavior, glial activity and cytokine levels are expected to revert to very low baseline (or close to baseline) levels after a transient immune challenge, even if there is a shift in the homeostatic baseline state (Figure 1).

**Figure 2:**
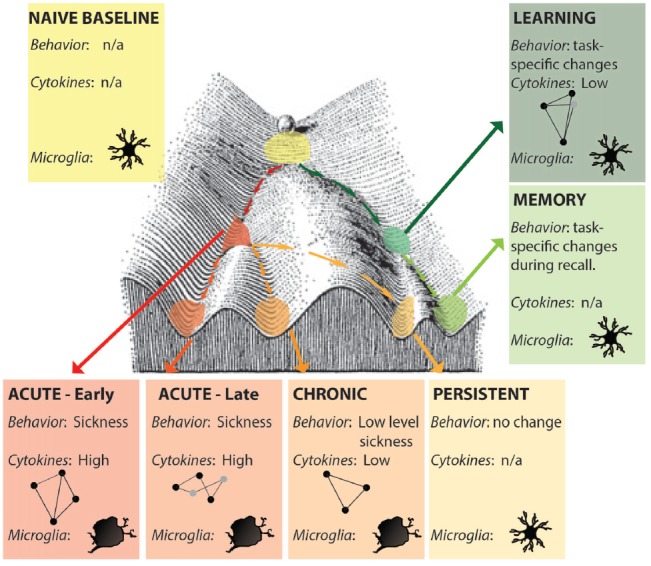
Distinct neuroimmune states. Each state is a unique snapshot of behavior, immune signaling, cellular changes, active cytokine networks and epigenetic signature at that time. Early- and late- acute immune states are distinguished by activation of different cytokine networks (represented by ball-and-stick figures); microglia show activated morphologies only during acute and chronic activation states; persistent changes are indistinguishable from naïve baseline in the absence of additional stimulation. Learning and memory may also be considered distinct neuroimmune (and epigenetic) states. Figure adapted from Waddington (1957).

Alternatively, defining an epigenetic landscape or signature has been one approach for defining states (Bonasio et al., 2010; Tronson et al., 2012; Nicol-Benoit et al., 2013). Here, unique epigenetic signatures of histone acetylation, phosphorylation and methylation and DNA methylation during different forms of neuroimmune activation, and that persist after resolution of immune signaling will better define the multiple acute and long-lasting brain states that can be induced by neuroimmune signaling (Figure 2). Indeed, epigenetic modifications and the resulting changes in gene expression profiles likely both define the state and drive the distinct patterns of signaling, behavior and cognitive changes that mediate.

### Implications of “Neuroimmune States” for Cognition and Behavior

There are several advantages to defining immune activation and post-activation as brain states. This view provides a framework to go beyond the idea of neuroimmune system as “on” or “off,” and towards an understanding of multiple active immune states such as those mediating different behavioral outcomes of bacterial or viral infections and provides a way to conceptualize sustained changes that persist long after resolution of acute neuroimmune activity.

There is clear evidence for roles of individual cytokines on cognition, memory and affective processes. Indeed, the activation of specific cytokine networks during learning suggests that, in addition to acute, chronic, and persistent states, neuroimmune signaling during learning and other cognitive tasks may be unique states themselves (Figure 2) that result in distinct epigenetic changes that mediate memory storage (Li et al., 2013). In addition, acute neuroimmune activation critically modulates memory and plasticity, yet depending on circumstances, it can either impair (Pugh et al., 1998; Goshen et al., 2007; Cross-Mellor et al., 2009; Cloutier et al., 2012) or enhance (Goshen et al., 2007; Mori et al., 2014; Delpech et al., 2015) memory and cognitive function. One important factor here is that the impact of immune signaling on cognition and behavior depends on the context of the specific immune milieu at this time (Becher et al., 2017). Indeed, in the periphery, determining the precise immune state can aid in minimizing side effects of immunologic treatments for systemic disease (Morel et al., 2017). By identifying distinct neuroimmune states, we will be better able to predict the effects of illness, immune activation, and specific cytokines on neuronal-glia interactions, plasticity and functional changes in cognition and behavior.

Finally, identifying persistent changes and shifts in the homeostatic baseline state as a consequence of illness or injury will provide a predictive marker for vulnerability or resilience to stress (Fonken et al., 2018), immune challenges (Norden et al., 2015; Muccigrosso et al., 2016), and neurodegeneration (Perry and Holmes, 2014; Hoeijmakers et al., 2016; McManus and Heneka, 2017) later in life. This is a particularly important avenue for research into individual differences and sex differences in susceptibility to immune-related disorders of cognition, memory and emotion (Perry et al., 2016; Snyder et al., 2016; Tronson and Collette, 2017; Choleris et al., 2018; Fisher et al., 2018; Speirs and Tronson, 2018).

## Author Contributions

DT and CP wrote the manuscript. NT developed the initial concept, wrote the discussion and edited the manuscript. All three authors contributed to the figures.

## Conflict of Interest Statement

The authors declare that the research was conducted in the absence of any commercial or financial relationships that could be construed as a potential conflict of interest.
